# Diffuse Coronary Vasospasm After Mitral Valve Replacement, Maze Procedure, and Tricuspid Valve Repair

**DOI:** 10.7759/cureus.35405

**Published:** 2023-02-24

**Authors:** Andrew J Gorton, Peter Rodgers-Fischl, John Gurley, Johannes Dorfling, Suresh Keshavamurthy

**Affiliations:** 1 Department of Surgery, Division of Cardiothoracic Surgery, University of Kentucky, Lexington, USA; 2 Department of Cardiovascular Medicine, University of Kentucky, Lexington, USA; 3 Department of Anesthesiology, University of Kentucky, Lexington, USA

**Keywords:** tricuspid valve, perioperative care, mitral valve replacement, coronary artery imaging, atrial fibrillation

## Abstract

Coronary vasospasm is a known complication after coronary artery bypass grafting surgery but has rarely been described in non-coronary cardiac operations. We report the case of a 51-year-old male with nonischemic cardiomyopathy and paroxysmal atrial fibrillation. He presented with severe mitral and tricuspid regurgitation and was taken for mitral valve replacement, tricuspid valve repair, and Maze procedure. Postoperative emergent coronary angiography demonstrated diffuse coronary vasospasm. Injection of intracoronary nitroglycerin led to clinical and angiographic improvement. This demonstrates the possibility of coronary vasospasm following mitral valve replacement and effective treatment with intracoronary administration of vasodilating agents.

## Introduction

Coronary vasospasm is a known and feared complication following coronary artery bypass grafting surgery that has rarely been described in non-coronary cardiac operations [1]. Iatrogenic causes of ST-segment changes, hemodynamic instability, or difficulty weaning from cardiopulmonary bypass (CPB) must be considered after valvular surgery and anti-arrhythmia procedures. Injury to the left circumflex artery during mitral valve operations and right coronary artery injury with the cryoprobe during Maze procedures are well known and carefully avoided [2,3]. However, there are few reports of coronary spasms following valve surgery. 

## Case presentation

**Figure 1:**
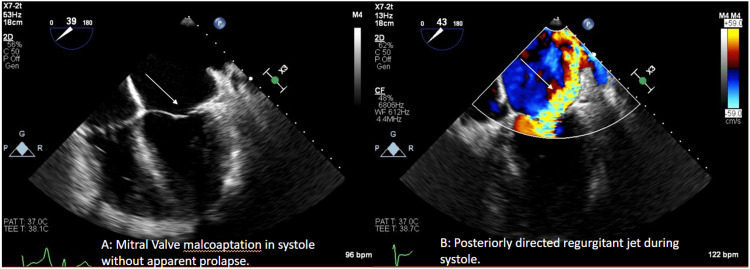
Preoperative Transesophageal Echocardiogram demonstrating severe Mitral Regurgitation due to systolic restriction of the anterior leaflet (A) with a posterior regurgitant jet (B). White arrows indicate findings noted.

**Figure 2:**
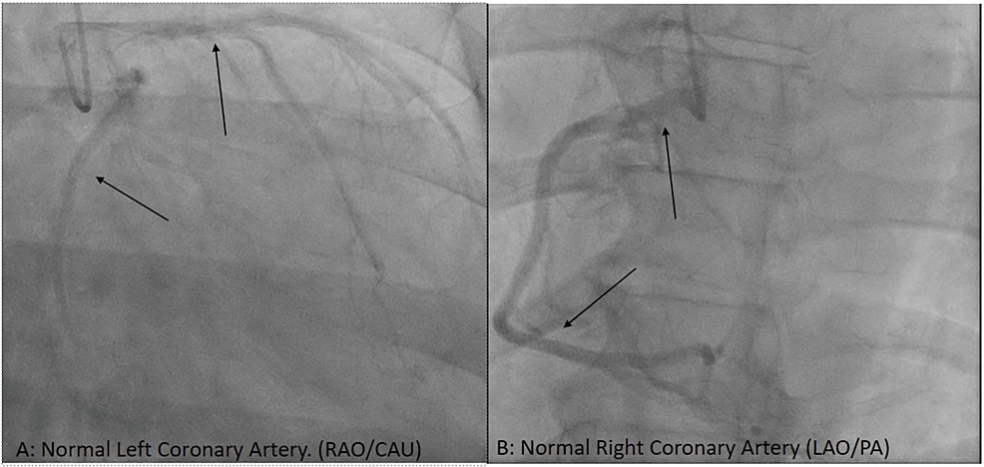
Preoperative coronary angiography demonstrating normal Left (A) and Right (B) coronary artery anatomy. Black arrows indicate representative segments.

We describe herein the case of a 51-year-old African American male with hypertension, paroxysmal atrial fibrillation, stage III chronic kidney disease, and nonischemic cardiomyopathy presenting with progressive shortness of breath and bilateral lower extremity edema. Echocardiogram demonstrated severe mitral regurgitation with asymmetric systolic restriction (Carpentier Type IIIc) and moderate-to-severe tricuspid regurgitation (Figure 1). Coronary angiography demonstrated normal right-dominant coronary anatomy (Figure 2). Right heart catheterization measured mean right atrial pressure of 19 mmHg, systolic pulmonary artery pressure of 67 mmHg, and mean pulmonary artery pressure of 49 mmHg. He elected for mitral valve replacement (MVR), tricuspid valve repair, Maze procedure (CMP), and left atrial appendage (LAA) clipping.

**Figure 3:**
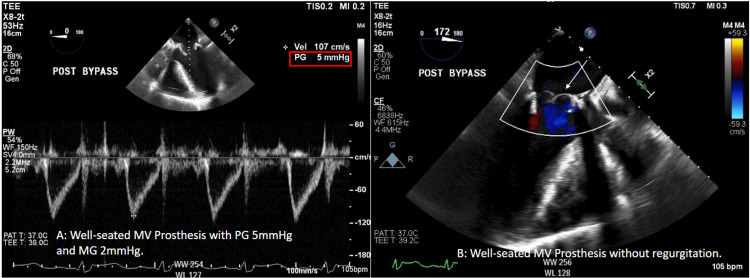
Post cardiopulmonary bypass transesophageal echocardiogram demonstrating a well seated mitral valve prosthesis with acceptable gradients (A) and no regurgitation or perivalvular leak (B). The red box and white arrow indicate relevant findings.

**Figure 4:**
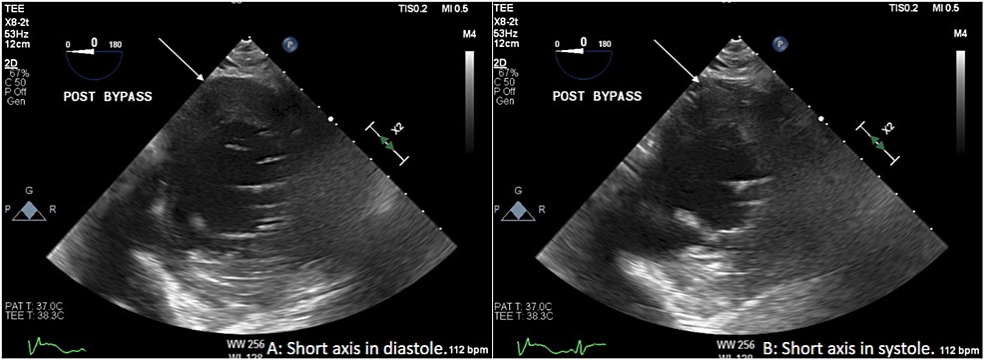
Post cardiopulmonary bypass transesophageal echocardiogram in short axis demonstrating dyskinesis of the inferior wall between diastole (A) and systole (B). White arrows identify an area of dyskinesis.

**Figure 5:**
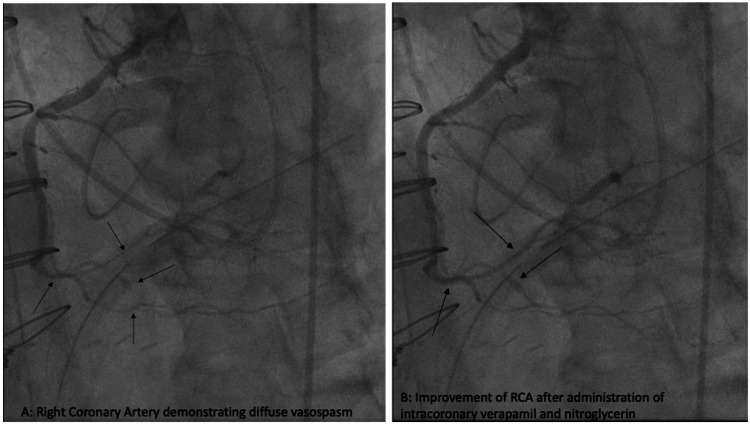
Coronary angiogram showing right coronary artery with diffuse spasm before (A) and after (B) intracoronary nitroglycerin administration.

**Figure 6:**
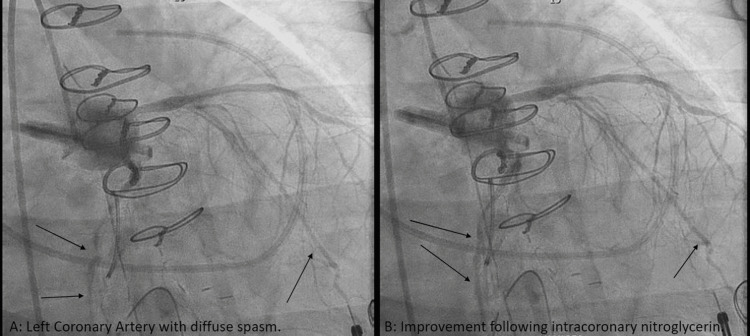
Coronary angiogram showing left coronary artery with diffuse spasm before (A) and after (B) intracoronary nitroglycerin administration.

Cardiopulmonary bypass (CPB) was initiated with Del Nido cardioplegia. The mitral valve was replaced with 33mm St. Jude Biocor Porcine bioprosthetic, tricuspid valve repair was performed with Edwards MC3 30mm annuloplasty ring, LAA was ligated with AtriCure 50mm AtriClip, and Cox-Maze IV was completed with a cryoprobe. The transesophageal echocardiogram showed a well-seated mitral valve prosthesis with a peak gradient of 5 mmHg, a mean gradient of 2 mmHg, and no paravalvular leak (Figure 3). While weaning from CPB, he developed intermittent T-wave elevations that resolved with increased systolic blood pressure to normal ranges. He was fully removed from CPB, hemostasis ensured, and the chest was closed with sternal wires. Following skin closure while still on the operating table, further ST-segment elevations were noted. Echocardiography demonstrated inferior wall dyskinesis without other wall motion abnormalities (Figure 4). Intravenous nitroglycerin and increased systolic blood pressure with norepinephrine failed to resolve the electrocardiographic changes. The patient was transported emergently to the cardiac catheterization laboratory, where coronary angiography demonstrated diffuse coronary spasm of both the right (RCA) (Figure 5A) and left coronary (LCA) systems (Figure 6A). Intracoronary nitroglycerin and verapamil were injected, resulting in an improvement in the angiographic appearance of the coronaries and resolution of the ST elevations (Figure 5B and Figure 6B). A nitroglycerin continuous infusion was started, and an intraaortic balloon pump (IABP) was placed to aid with the weaning of vasoactive agents. On postoperative day two, the IABP was removed, and the nitroglycerin infusion was weaned. He had no further electrocardiographic changes.

## Discussion

Coronary vasospasm is a known and feared complication after coronary artery bypass grafting surgery but has rarely been described in non-coronary cardiac operations. Iatrogenic causes of ST-segment changes, hemodynamic instability, or difficulty weaning from CPB must be considered after valvular surgery and anti-arrhythmia procedures. Left circumflex artery injury or occlusion during mitral valve surgery and right coronary artery injuries during tricuspid valve operations are well described [2,3]. However, there are few reports of coronary spasms following valve surgery, Maze procedure, or combined operations. 

H Shafei et al. [4] reported on a 50-year-old female with the inability to wean from CPB following MVR. On examination, her anterolateral left ventricular wall was akinetic, and a vein graft was used to bypass the left anterior descending artery. Coronary spasm was identified three months postoperatively on provocative testing during coronary arteriography. C Pragliola et al. [5] presented a 67-year-old male that developed focal RCA spasm after MVR. Intracoronary nitrate injection relieved the spasm and improved inferior wall cardiac function. M Kanno et al. [6] described a 50-year-old female undergoing reoperative mitral valve replacement that developed a right coronary artery spasm leading to ischemia. This was treated with intravenous diltiazem and confirmed on coronary angiography. T Ahmad et al. [7] reported a 63-year-old male undergoing AVR and MVR with diffuse spasm of the entire coronary tree four hours postoperatively. Coronary angiography identified the pathology, and intracoronary nitroglycerin injection relieved the spasm. Q Lang et al. [8] described the case of a 66-year-old male undergoing MVR and CMP for severe mitral stenosis and persistent atrial fibrillation. Four hours after an uneventful operation, he developed inferior lead electrocardiographic changes, and coronary angiography identified a distal RCA spasm which was relieved with intracoronary nitroglycerin injection. BG Rajbanshi et al. [9] described a 45-year-old female that developed focal RCA spasm following CMP diagnosed on coronary angiography and reversed with intracoronary nitroglycerin. 

Unlike most of the cases above, our patient developed ST segment changes while still in the operating room. The decision to pursue coronary angiography rather than reopening and performing a bypass graft is not trivial, but neither is the exercise of attempting a graft to the obtuse marginal branches. We must also consider the potential injury from cryoablation used during the CMP. Direct ablation of the coronary arteries may lead to significant obstruction or total occlusion. There is also a theoretical risk of indirect injury from cryolesions penetrating too deeply or pericoronary edema following the procedure. If technical considerations have been ruled out as a source of coronary injury, systemic administration of vasodilators is an appropriate next step. If this fails to address the issue, then in the appropriate patient, transportation to the coronary catheterization laboratory can provide further information via emergent coronary angiography with the potential to deliver intracoronary vasodilators and perform other advanced interventions. This capability does require a close working relationship between the surgical, anesthesia, and interventional teams within the hospital system. The availability of interventional cardiology allowed us to avoid a bypass graft that likely would have failed in the short term and not addressed the underlying pathology. 

## Conclusions

Diffuse coronary vasospasm is a rare and potentially devastating complication following noncoronary cardiac surgery that should be considered in the setting of ECG changes and hemodynamic instability. As demonstrated by this case and the others cited, coronary spasms may present intraoperatively, shortly after the patient arrival to the intensive care unit, or in the first days after noncoronary surgery. Consideration of this pathology in the event of new ECG changes and hemodynamic changes will allow for timely diagnosis and treatment.

## References

[1] He GW, Taggart DP (2016). Spasm in arterial grafts in coronary artery bypass grafting surgery. Ann Thorac Surg.

[2] Bargagna M, Trumello C, Sala A, Blasio A, Castiglioni A, Alfieri O, De Bonis M (2021). Left circumflex artery injury after mitral valve surgery: An algorithm management proposal. Ann Thorac Surg.

[3] Gerçek M, Omran H, Friedrichs KP (2022). Right coronary artery deformation and injury following tricuspid valve surgery. Front Cardiovasc Med.

[4] Shafei H and Bennett JG (1990). Coronary artery spasm during mitral valve replacement. Eur J Cardio-thorac Surg.

[5] Pragliola C, Gaudino M, Farina P, Massetti M (2015). Postoperative coronary artery spasm after mitral valve replacement. Int J Surg Case Rep.

[6] Kanno M, Kurihara H, Satoh H, Hamawaki M, Honda M (1994). [Coronary artery spasm after mitral valve replacement: a case report]. Kyobu Geka.

[7] Ahmad T, Kishore KS, Maheshwarappa NN, Pasarad AK (2015). Postoperative diffuse coronary spasm after two valve surgery - a rare phenomenon. Indian Heart J.

[8] Lang Q, Li C, Qin C, Meng W (2021). Postoperative coronary artery spasm after mitral valve replacement and cox-maze IV procedure: A case report. Heart Surg Forum.

[9] Rajbanshi BG, Rodrigues E, Lynch JJ, Gulati R, Sundt TM 3rd (2011). Coronary artery spasm after cryo maze III procedure. Ann Thorac Surg.

